# Incorporating the anterior mitral leaflet to the annulus abolishes leaflet movement, the diastolic baffle effect, and shortens the long axis of the ventricle

**DOI:** 10.1016/j.xjon.2021.07.009

**Published:** 2021-07-17

**Authors:** Laurencie Brunel, Hugh S. Paterson, Paul G. Bannon

**Affiliations:** a Faculty of Sciences, School of Veterinary Sciences, The University of Sydney, Sydney, Australia; b DVC Research Portfolio, The University of Sydney, Sydney, Australia; c Institute of Academic Surgery, Royal Prince Alfred Hospital, Camperdown, Australia; d Faculty of Medicine and Health, Central Clinical School – Surgery, The University of Sydney, Sydney, Australia

To the Editor:

**Figure:**
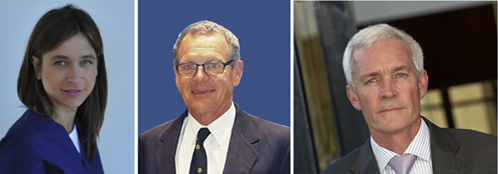

The authors reported no conflicts of interest.

The *Journal* policy requires editors and reviewers to disclose conflicts of interest and to decline handling or reviewing manuscripts for which they may have a conflict of interest. The editors and reviewers of this article have no conflicts of interest.

We thank Dr Timek for his comments regarding the elegant approach to investigating the implications of incorporating (reefing) the anterior mitral leaflet to the anterior annulus.^1^^,^^2^ Dr Timek noted that the reefing intervention abolished anterior leaflet movement, distorted left ventricular geometry by a reduction in anterior leaflet height, and removed the baffle effect that promotes laminar blood flow through the ventricle during diastole. The effects of these components on left ventricular function are difficult to assess independently but as a whole, there was impairment of left ventricular function.

We wish to clarify the issues that Dr Timek has raised with regard to left ventricular outflow tract obstruction and aortic valve incompetence. The pressure components of the hemodynamic parameters altered by the reefing intervention were measured with a solid-state pressure catheter in the left ventricle. We have demonstrated in a separate sheep model that left ventricular outflow tract obstruction causes an increase in intraventricular pressure rather than a decrease.^3^ A separate pressure catheter was positioned in the aortic arch and the pressure continuously observed and recorded throughout each study. Any “subclinical” outflow tract obstruction did not manifest as an outflow tract gradient.

Both reefing the anterior leaflet and insertion of the mitral prosthesis can cause aortic valve incompetence. We performed a detailed echocardiographic assessment at the time of initial reefing and releasing of the anterior leaflet in each sheep. This included an assessment of the left ventricular outflow tract. We did not repeat this assessment for subsequent interventions in the absence of suspicion based on altered hemodynamic parameters.

We hope that our findings do add to the basic understanding of mitral valve physiology and acknowledge that there are limitations to the clinical extrapolation. We remain grateful to Dr Timek for his leadership in this field of research.
